# MRS suggests multi-regional inflammation and white matter axonal damage at 11 years following perinatal HIV infection

**DOI:** 10.1016/j.nicl.2020.102505

**Published:** 2020-11-19

**Authors:** Amy S. Graham, Martha J. Holmes, Francesca Little, Els Dobbels, Mark F. Cotton, Barbara Laughton, Andre van der Kouwe, Ernesta M. Meintjes, Frances C. Robertson

**Affiliations:** a Biomedical Engineering Research Centre, Division of Biomedical Engineering, Department of Human Biology, University of Cape Town, South Africa; b Neuroscience Institute, University of Cape Town, South Africa; c Department of Statistical Sciences, University of Cape Town, South Africa; d Family Centre for Research with Ubuntu, Department of Paediatrics & Child Health, Stellenbosch University, South Africa; e A.A. Martinos Centre for Biomedical Imaging, Massachusetts General Hospital, Boston, USA; f Department of Radiology, Harvard Medical School, Boston, USA; g Cape Universities Body Imaging Centre, University of Cape Town, South Africa

**Keywords:** Antiretroviral therapy, Basal ganglia, HIV, Midfrontal gray matter, Peritrigonal white matter, Proton magnetic resonance spectroscopy

## Abstract

The neurological changes in children living with perinatal HIV (PHIV) on antiretroviral therapy (ART) can be studied at a metabolic level through proton magnetic resonance spectroscopy. While previous studies in children have largely focused on individual metabolite changes, investigating patterns within and across regions of interest can aid in identifying metabolic markers of HIV infection.

In this study 76 children with PHIV from the Children with HIV Early AntiRetroviral (CHER) trial, 30 children who were HIV-exposed-uninfected (HEU) and 30 children who were HIV-unexposed (HU), were scanned at the age of 11.6 (sd = 0.3) years using a 3 T Skyra scanner. Metabolite concentrations were quantified within the basal ganglia (BG), midfrontal gray matter (MFGM) and peritrigonal white matter (PWM), comparing levels between HIV status groups using linear regression. Factor analysis and logistic regression were performed to identify metabolic patterns characteristic of HIV infection within and across the regions of interest.

In the BG region we observed restored metabolic activity in children with PHIV and children who were HEU, despite differences being previously observed at younger ages, suggesting that treatment may effectively reduce the effects of HIV infection and exposure. Elevated MFGM choline levels in children with PHIV are indicative of inflammation. Further, we observed reduced N-acetyl-aspartate (NAA) in the PWM of children with PHIV and children who were HEU, indicating possible axonal damage. Lower levels of PWM creatine in children with PHIV suggest that this may not be a valid reference metabolite in HIV studies. Finally, factor scores for a cross-regional inflammatory factor and a PWM axonal factor, driven by PWM NAA and creatine levels, distinguished children with PHIV from children without HIV (HEU and HU) at 11 years. Therefore, the effects of perinatal HIV infection and exposure continue to be seen at 11 years despite early treatment.

## Highlights

• Elevated gray matter choline suggests inflammation in children with PHIV.
• Children with PHIV and exposure have lower white matter NAA, implying axonal damage.
• A multi-regional inflammatory factor differs between children with and without PHIV.
• Absolute metabolite concentrations should be considered along with creatine ratios.

## 1 Introduction

Despite initiatives that have improved antiretroviral therapy (ART) accessibility for pregnant women living with HIV (WLHIV), nine percent of infants born to WLHIV in Southern and Eastern Africa in 2018 were estimated to have contracted HIV (UNAIDS, 2019). Infants still acquire perinatal and postnatal HIV infection due to various factors including premature birth, issues of adherence and ART not providing complete protection (Stringer et al., 2010, Warszawski et al., 2008).

The Children with HIV Early AntiRetroviral (CHER) trial was established to determine when to initiate treatment in infants living with perinatal HIV (PHIV) in South Africa (Cotton et al., 2013, Violari et al., 2008). This trial highlighted the value of early treatment in infants under 12 weeks of age, reducing mortality rates and morbidity, as seen by improved short-term cognition and health status (Cotton et al., 2013, Laughton et al., 2012, Violari et al., 2008). Based on the findings of various clinical trials, guidelines for treating children with PHIV prioritise immediate treatment in children below the age of 5 years following a positive diagnosis (WHO, 2017, WHO, 2013).

ART is a life-long necessity for persons living with HIV, as viral reservoirs enable the pathogens to persist within their host, leading to a resurgence of infection if treatment is discontinued (Joos et al., 2008). Despite early ART and viral suppression, children with PHIV from the CHER trial continue to demonstrate subtle cognitive deficits (Laughton et al., 2018), as well as brain functional and structural abnormalities (Toich et al., 2018, Jankiewicz et al., 2017, Randall et al., 2017, Ackermann et al., 2016). Moreover, in animal studies, detrimental neurological effects have been demonstrated from antiretroviral drugs themselves (Robertson et al., 2012).

The introduction of effective ART for pregnant WLHIV has, however, led to a reduction in the vertical transmission of HIV to neonates (UNAIDS, 2019, De Cock et al., 2000). Thus, there is a growing population of children who were perinatally HIV-exposed-uninfected (HEU), who may exhibit developmental delays (Van Rie et al., 2008), and/or low visual IQ and attention scores (Kerr et al., 2014). HIV exposure has also been associated with mental health disorders, such as behavioural or mood issues and anxiety, possibly with higher frequency than in children with HIV receiving interventions (Mellins et al., 2012, Malee et al., 2011).

Single voxel proton magnetic resonance spectroscopy (^1^H-MRS) examines the underlying metabolic changes in select brain regions in a non-invasive way (Wilkinson et al., 1997) and may be more sensitive for detecting subtle changes in the brain in people with ART-induced viral suppression, compared to assessing structural or functional differences (Paul et al., 2008). The metabolites of interest in MRS studies include the excitatory neurotransmitter glutamate (Glu), creatine (Cr), an essential metabolite for energy generation, and N-acetyl-aspartate (NAA), an indicator of neuronal integrity (for review see Soares and Law, 2009). Choline (Cho), a cell membrane component, and myoinositol (Ins), a marker of glial cells, can also be quantified. Total choline (glycerophosphocholine + phosphocholine = tCho), total creatine (creatine + phosphocreatine = tCr) and total NAA (NAA + N-acetyl-aspartyl-glutamate = tNAA) can be measured.

We sought to examine differences in metabolic activity at 11 years in three brain regions of interest – the basal ganglia (BG), midfrontal gray matter (MFGM) and peritrigonal white matter (PWM) – in a subset of children from the CHER trial and in children who were HEU and HIV-unexposed (HU). The BG, a notable target of HIV, is involved in memory processes (for review see Graybiel, 1995), language interpretation (Booth et al., 2007) and in signalling pathways of the frontal lobe that control motor skill activity and behaviour (Bonelli and Cummings, 2007, Alexander et al., 1986). The MFGM, representing the last brain lobe to develop (Casey et al., 2008), has important roles in cognitive activity (Diamond, 2002) and decision-making (Brass and Von Cramon, 2002). Finally, PWM is a region of late myelination (Parazzini et al., 2002) in which children with PHIV from the CHER trial were previously found to display structural abnormalities (Ackermann et al., 2014).

Altered neurometabolic activity was noted in these children in the selected regions at younger ages. Elevated NAA and tCho were detected in the BG at 5 years in children with PHIV placed on treatment within the first 12 weeks after birth, compared to children without HIV – the majority of whom were HIV exposed (Mbugua et al., 2016). While children with PHIV and children who were HEU showed similar metabolic levels to controls in the BG at age 7 years, reduced NAA and Glu were evident in both groups at 9 years, as well as children who were HEU expressing lower tCr and tCho (Robertson et al., 2018).

As metabolic activity changes with age (Holmes et al., 2017), the present study sought to cross-sectionally assess the long-term effects of perinatal HIV infection in the presence of early ART, and the impact of HIV exposure, on neurometabolic activity at 11 years. Adding to previous cross-sectional evaluations of children with PHIV from the CHER trial, who were placed on the same ART regimen before the age of 2 years, this study sought to examine whether the combined effects of HIV and ART would persist, specifically as the children enter pre-adolescence, or if metabolic activity would be restored. Additionally, we aimed to investigate interregional and intraregional metabolite relationships (as in Yiannoutsos et al., 2004) to identify patterns of metabolic activity that distinguish children with PHIV from children without HIV (HEU and HU) at 11 years. Based on earlier findings (Robertson et al., 2018), we hypothesised that children with PHIV would have lower NAA and Glu.

## 2 Methods

### 2.1 Participants

Of 136 children in this study, 76 were children with PHIV who had been enrolled in the CHER trial, in which asymptomatic infants age 6–12 weeks with CD4 percentages ≥ 25%, were randomised to three treatment groups (Violari et al., 2008). Two groups were placed on early time-limited treatment (initiated before 12 weeks old) for 40 or 96 weeks, respectively, while treatment was delayed in the third group until a clinical event occurred or CD4 percentage dropped below 20%, or below 25% if younger than 12 months (Cotton et al., 2013, Violari et al., 2008). The same criteria were used to reinitiate ART in the early treatment groups. All children in our sub-sample initiated treatment by 19 months of age. Viral load was suppressed in 61% of children by the age of 1 year and 74% by the age of 18 months (N = 72). At the time of their 11-year scan, 97% were virally suppressed, having achieved viral suppression at an earlier time point. The first-line ART regimen consisted of lamivudine & zidovudine (ViiV) and lopinavir-ritonavir (Violari et al., 2008).

At the time of the 11-year scan, 58 children with PHIV were on an unchanged regimen, while in eight children abacavir replaced zidovudine. Five children were on lamivudine, abacavir and efavirenz, two children were taking lamivudine, zidovudine and efavirenz and one child was on emtricitabine/tenofovir alafenamide fumarate. Two children were on different combinations of four different drugs – zidovudine or abacavir with darunavir, ritonavir and raltegravir due to failing 2 previous regimens.

30 children who were HEU, born to WLHIV and 30 children who were HU, initially enrolled in a pneumococcal vaccine trial (Madhi et al., 2010) or enrolled later from similar communities, also participated in the study.

Ethical approval for this study was provided by the Human Research Ethics Committees of the Faculties of Health Science at both the Universities of Cape Town and Stellenbosch. Parents provided written informed consent and children assent.

### 2.2 Neuroimaging

**Fig. 1:**
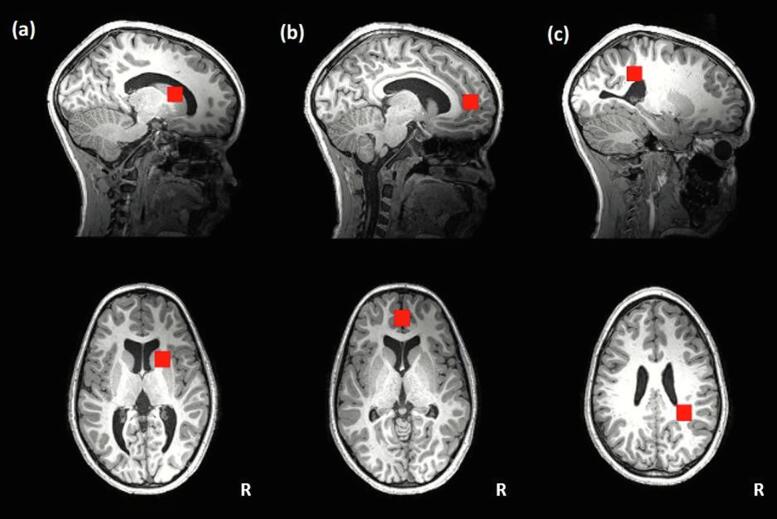
Sagittal and axial views of the (a) basal ganglia, (b) midfrontal gray matter and (c) peritrigonal white matter regions (1.5×1.5×1.5 cm^3^) in which MR spectroscopy data were acquired.

The children were scanned at a mean age of 11.6 (sd = 0.3) years at the Cape Universities Body Imaging Centre (CUBIC) at the University of Cape Town, using a 3 T Skyra scanner (Siemens, Erlangen, Germany). Structural magnetic resonance imaging (MRI) scans were obtained using a multi-echo magnetization prepared rapid gradient echo (MEMPRAGE) sequence (van der Kouwe et al., 2008). Pulse sequence timings were as previously described (Robertson et al., 2018)*,* with an inversion time (TI) of 1100 ms, repetition time (TR) of 2530 ms, echo times (TE) of 1.69/3.54/5.39/7.24 ms, a resolution of 1.0×1.0×1.0 mm^3^ and 176 slices. Single voxel ^1^H-MRS was acquired in three regions of interest (1.5x1.5x1.5 cm^3^) in the brain: the BG, MFGM and PWM (Fig. 1). A point resolved spectroscopy (PRESS) sequence was utilised (TR 2000 ms, TE 30 ms, 1300 Hz bandwidth), using chemical shift selective (CHESS) water suppression with 64 averages. A single water reference scan was obtained without water suppression.

### 2.3 Processing of spectra

Structural MR images were processed using Statistical Parametric Mapping (SPM12) (http://www.fil.ion.ucl.ac.uk/spm) to characterise the gray matter (GM), white matter (WM) and cerebrospinal fluid (CSF) content of the regions of interest (Supplementary Table 1). Eddy current and baseline signal corrections were performed in LCModel (version 6.3-1), whereafter the water-scaling approach was used to obtain absolute metabolite concentrations, as well as ratios to total creatine (tCr) (Helms, 2008, Provencher, 2001, Ernst et al., 1993).

Spectral quality was assessed in terms of the signal-to-noise ratio (SNR) and the full width at half maximum (FWHM). Scans not meeting the criteria of SNR > 7.0 and FWHM < 0.07 ppm were excluded from further analyses (as in Robertson et al., 2018). Outliers, defined as values >1.5 times the interquartile range above or below the upper and lower quartiles respectively (Horn et al., 2001), were excluded if they altered the results.

### 2.4 Statistical analysis

Linear regression analysis was carried out in R (R Core Team, 2018) to compare absolute concentrations of metabolites, as well as ratios to tCr, in children with PHIV and children who were HEU to children who were HU. For each metabolite, HIV status groups were compared within a single linear regression model. Sex, GM or WM content and age at scan were controlled for as confounders (see S.2.1 of appendix). Although Bonferroni correction for the 33 tests performed would require an unadjusted p < 0.0015 (0.05/33) for significance, we focus on results at a 5% level of significance with unadjusted p < 0.05 as a way of highlighting the stronger effects. These results are viewed in an explanatory manner and do not attempt to confirm a hypothesis. Hence emphasis is not placed on statistical significance. Findings with p-values between 0.05 and 0.1, albeit beyond the conventional threshold of significance, are additionally reported and discussed.

A further comparison between children with PHIV and children without HIV (HEU and HU) was made using factor analysis. Factor analysis including five metabolites, tCho, tCr, tNAA, Ins and Glu, across the three regions of interest resulted in region-specific metabolite groupings (data not shown). Three metabolites, tCho, tNAA and tCr in each of the three regions, were selected for further factor analysis to investigate the interaction of these metabolites across regions, due to their more robust measurements (low % standard deviation) and their association with HIV infection.

Metabolites were mean-centered and scaled. The maximum likelihood method was used for factor extraction followed by orthogonal (*varimax*) rotation (Kaiser, 1958). To determine the number of factors to retain, we required the low dimensional approximation to account for at least 70% of the total variability in the data. Factor scores were calculated for each of the factors using a weighted scoring approach. In this approach, the relative weightings for all metabolites contributing to a given factor, determined using factor loadings, are summed and then divided by the sum of the weights, to give the factor score (as in Yiannoutsos et al., 2004). Names were assigned to each factor based on the main contributing metabolites, their roles and physiological indications.

Factor scores were included in logistic regression models comparing children with PHIV versus HEU and HU combined, adjusting for sex, age at scan, GM and WM content, retaining only factor scores that were associated with the outcome in the final model (see S.2.2 of appendix). A receiver operating characteristic (ROC) curve was generated to determine the predictive capability of this model.

## 3 Results

### 3.1 Summary of participant demographics

Table 1 gives a summary of the demographics for the children at 11 years. One-way analysis of variance (ANOVA) with post-hoc pairwise group comparisons, showed that children who were HEU were, on average, about 1 month and 2 months younger than children with PHIV and children who were HU, respectively (p = 0.004 and 0.002). At the time of scanning, children with PHIV weighed less and were shorter than children who were HU (p = 0.006 and p = 0.008). Statistical analysis of the proportion of males in each group did not yield a meaningful difference between the groups.

**Table 1:** Sample demographics.

N = 136	PHIV	HEU	HU	χ^2^ or F (p)
n	76	30	30	
Sex (Male)	36 (47%)	20 (67%)	15 (50%)	3.3 (0.19)
Age at scan (years)	11.6 (0.3)	11.5 (0.2)	11.7 (0.2)	5.9 (0.004)
Weight at scan (kg)	35.2 (8.2)	38.1 (11.8)	41.1 (11.1)	4.1 (0.02)
Height at scan (cm)	140.0 (9.0)	142.6 (10.2)	145.5 (8.9)	3.7 (0.03)

Values are mean (standard deviation); PHIV: children with perinatal HIV; HEU: HIV-exposed-uninfected; HU: HIV-unexposed.

Quality checks led to three children being excluded from analyses of BG spectra (1 PHIV, 1 HEU and 1 HU), while four children were excluded from analysis of MFGM (3 PHIV and 1 HU) and seven were excluded for the PWM spectra (3 PHIV, 1 HEU and 3 HU). These children were all excluded from both factor analysis and logistic regression analysis.

A summary of the clinical measures at enrollment and around the time of scan is provided in Table 2. The average age at which children began ART was 14.7 weeks. All the children with PHIV began treatment before 76 weeks of age, with treatment being interrupted as per the CHER trial protocol in 38 children – 23 around age 40 weeks and 15 around age 96 weeks.

**Table 2:** Clinical measures for children with PHIV.

ART initiation and interruption	N = 76
Age at starting ART (weeks)	14.7 (sd = 13.4) range 5.9–75.7
Children with interrupted treatment	38 (50%)
**Age at interruption (weeks)**	
Around 40 weeks (N = 23)	49.6 (sd = 2.7) range 45–56
Around 96 weeks (N = 15)	105.4 (sd = 3.2) range 95–115
Duration of interruption (weeks)	80.4 (sd = 103.0) range 5.7–398.0
**At enrollment to CHER trial**	
CD4 count (cells/mm^3^)	1819 (sd = 894)
CD4%	32.9 (sd = 10.5)
CD4/CD8 *	1.3 (sd = 0.7)
Baseline viral load **	
High	41 (57%)
Low	31 (43%)
Suppressed	0
**At scan**	
CD4 count (cells/mm^3^) ***	900 (sd = 360)
CD4% ***	37.8 (sd = 6.8)
Viral load at scan ****	
High	0
Low	2 (3%)
Suppressed	69 (97%)
**CDC classification**	
A	8 (11%)
B	14 (18%)
Severe B	14 (18%)
C	40 (53%)

Viral load categories: high (>750,000 copies/mL), low (between 400 and 750,000 copies/mL), suppressed (<400 copies/mL).

* CD8 data at enrollment obtained after 12 weeks for 10 children and missing for 3 children.

** VL at enrollment missing for 4 children.

*** CD4 data for 8 children were excluded as they were > 6 months from scan.

**** VL data at scan for 5 children were > 6 months from scan and thus excluded.

The GM, WM and CSF fraction of the BG and PWM were similar between HIV status groups, while the MFGM composition differed between these groups (Supplementary Table 1). Specifically, children with PHIV had a lower proportion of WM in comparison to children who were HEU (p = 0.014).

Analyses comparing CD4 counts and CD4 percentages at enrollment and at scan, and CD4/CD8 ratios at enrollment, between children with PHIV in whom treatment had been interrupted and those on continuous treatment, yielded no differences.

### 3.2 Linear regression analysis

No clear differences in BG metabolite levels were observed between the three HIV status groups (Table 3, Table 4). However, children with PHIV demonstrated higher tCho/tCr compared to controls that fell short of conventional levels of significance (Table 4), despite showing no evidence of higher absolute tCho levels (Table 3). Children who were HEU demonstrated lower Ins in the BG that fell short of conventional levels of significance compared to children who were HU (Table 3).

**Table 3:** Unstandardized coefficients (B), standard errors (SE) and p-values (p) from linear regression analyses comparing absolute metabolite concentrations in the basal ganglia, midfrontal gray matter and peritrigonal white matter of (left) children with perinatal HIV (PHIV) and (right) children who were HIV-exposed-uninfected (HEU), to children who were HIV-unexposed (HU). Age at scanning, sex and gray or white matter content have been adjusted for.

	PHIV			HEU		
	B	SE	p	B	SE	p
**Basal Ganglia** (N = 133: 75 PHIV, 29 HEU, 29 HU)						
Total NAA	−0.123	0.159	0.439	−0.262	0.194	0.181
NAA	−0.232	0.147	0.118	−0.241	0.182	0.187
Glu	0.139	0.207	0.502	0.157	0.251	0.534
Total Cho	0.051	0.033	0.131	−0.057	0.041	0.167
Ins	0.049	0.137	0.718	−0.303	0.170	0.076
Total Cr	−0.070	0.119	0.558	−0.118	0.145	0.419
**Midfrontal gray matter** (N = 132: 73 PHIV, 30 HEU, 29 HU)						
Total NAA	0.062	0.153	0.684	−0.307^a^	0.190	0.108
NAA	−0.073^b^	0.135	0.590	**−0.347**^a^	**0.165**	**0.038**
Glu	−0.274^b^	0.214	0.204	**−0.529**^a^	**0.266**	**0.048**
Total Cho	**0.127**	**0.037**	**<0.001**	−0.022^a^	0.046	0.635
Ins	0.253	0.132	0.057	−0.288^a^	0.163	0.079
Total Cr	0.098	0.113	0.387	−0.168^a^	0.139	0.227
**Peritrigonal white matter** (N = 129: 73 PHIV, 29 HEU, 27 HU)						
Total NAA	**−0.370**	**0.173**	**0.034**	**−0.491**	**0.213**	**0.023**
NAA	−0.312	0.166	0.063	**−0.414**	**0.203**	**0.044**
Glu	−0.297^c^	0.183	0.107	−0.249^c^	0.222	0.263
Total Cho	0.005	0.036	0.900	−0.052	0.044	0.244
Ins	−0.247	0.130	0.061	−0.240	0.159	0.132
Total Cr	**−0.166**	**0.074**	**0.027**	−0.118	0.091	0.199

a One HEU child was excluded from all midfrontal gray matter analyses due to having influential results across all metabolites, as shown in Fig. 2.

b One PHIV and one HU outlier excluded from midfrontal gray matter NAA analysis.

c 1 PHIV and 1 HU outlier excluded for peritrigonal white matter Glu analysis; Bold denotes p < 0.05, unadjusted for multiple comparisons.

**Table 4:** A summary of unstandardized coefficients (B), standard error (SE) and p values (p) for linear regression analyses assessing the ratios of absolute metabolite concentrations to total creatine (tCr) in the basal ganglia, midfrontal gray matter and peritrigonal white matter in, respectively, (left) children with perinatal HIV (PHIV) and (right) children who were HIV-exposed-uninfected (HEU) compared to children who were HIV-unexposed. Age at scan, sex, and gray or white matter content have been adjusted for.

	PHIV			HEU		
	B	SE	p	B	SE	p
**Basal Ganglia**						
Total NAA/tCr	−0.004	0.019	0.843	−0.020	0.024	0.392
NAA/tCr	−0.018	0.018	0.336	−0.019	0.022	0.392
Glu/tCr	0.033	0.026	0.213	0.040	0.032	0.213
Total Cho/tCr	0.007^a^	0.004	0.064	−0.006	0.005	0.225
Ins/tCr	0.009	0.017	0.585	−0.036	0.021	0.098
**Midfrontal gray matter**						
Total NAA/tCr	−0.003	0.019	0.860	−0.014	0.023	0.546
NAA/tCr	−0.005	0.019	0.804	−0.015	0.023	0.526
Glu/tCr	−0.032	0.032	0.316	−0.020	0.039	0.615
Total Cho/tCr	**0.015**	**0.005**	**0.002**	0.002	0.006	0.757
Ins/tCr	0.026	0.015	0.094	−0.018	0.019	0.328
**Peritrigonal white matter**						
Total NAA/tCr	−0.012	0.034	0.733	−0.062	0.042	0.139
NAA/tCr	−0.011	0.031	0.727	−0.051	0.038	0.183
Glu/tCr	−0.032	0.042	0.451	−0.031	0.051	0.545
Total Cho/tCr	0.012	0.008	0.110	−0.002	0.010	0.809
Ins/tCr	−0.018	0.028	0.526	−0.027	0.034	0.418

a 1 PHIV outlier removed from analysis of tCho/tCr in the Basal Ganglia. Bold denotes p < 0.05, unadjusted for multiple comparisons.

In the MFGM the absolute concentration of tCho was higher in children with PHIV than children who were HU (Table 3; Fig. 2a). Similarly, when looking at the ratio of metabolites to tCr, increases in tCho/tCr were observed in the MFGM (Table 4). Children with PHIV additionally tended to have higher levels of absolute Ins in the MFGM that fell just short of conventional levels of significance (Table 3). Children who were HEU demonstrated lower absolute levels of NAA and Glu in the MFGM and tended to have lower Ins, albeit outside conventional significance levels, compared to controls (Table 3; Fig. 2 b & c).

**Fig. 2:**
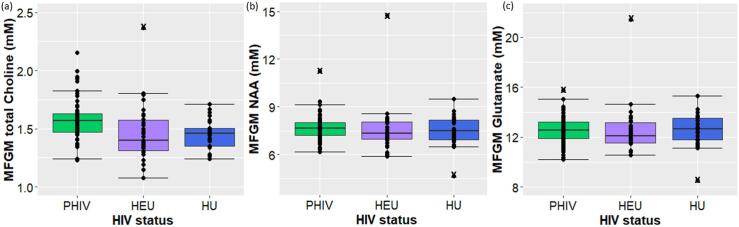
Dot and box plots (median and IQR) showing the absolute concentrations of (a) total choline (b) NAA and (c) glutamate in the midfrontal gray matter at 11 years, according to HIV status group (73 PHIV, 30 HEU, 29 HU). Outliers excluded from analyses are indicated with an X.

**Fig. 3:**
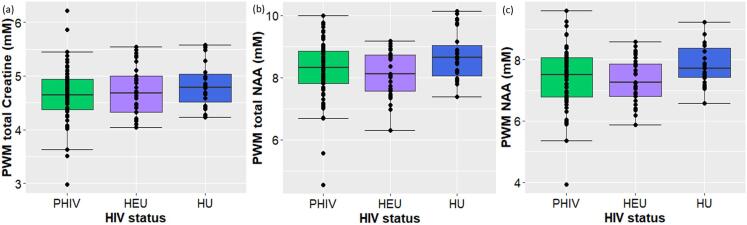
Box and dot plots (median and IQR) showing the absolute concentrations of (a) total creatine (b) total NAA and (c) NAA in the peritrigonal white matter (PWM) according to HIV status at the age of 11 years (73 PHIV, 29 HEU, 27 HU).

In the PWM, by contrast, children with PHIV showed a reduction in the absolute concentrations of tCr and tNAA (Table 3 Fig. 3 a & b) and, although being above the threshold of significance, tended to have lower levels of NAA and Ins compared to children who were HU (Table 3). Children who were HEU also had lower levels of tNAA and NAA than children who were HU (Table 3 Fig. 3 b & c). Analysis of the ratios of the various metabolites to tCr, in this case, yielded no notable differences between the HIV status groups (Table 4).

In a comparison between children with PHIV in whom treatment had been interrupted and those on continuous treatment, we found no differences for tCho in the MFGM or tCr and tNAA in the PWM. However, when comparing these groups to children who were HU, tCr in the PWM was lower only in children with PHIV who had uninterrupted treatment (p = 0.021).

Elevated tCho in the MFGM of children with PHIV compared to unexposed controls, was the only result that remained at the new threshold of significance (p < 0.0015) after correction for multiple comparisons, while the finding of a higher ratio of tCho/tCr in the same region fell just short of this adjusted level of significance.

### 3.3 Factor analysis and logistic regression analysis

Factor analysis with *varimax* rotation, resulting in factors being perpendicular to each other, involved the three metabolites of interest across three regions. This generated five factors which accounted for 71.0% variation in the data (Table 5). These factors include a factor driven by tCho in each of the regions (Multi-regional inflammatory factor), two factors in which tNAA and tCr are grouped within the PWM and MFGM (the PWM axonal and MFGM neuronal factors, respectively), and finally two factors separately driven by tCr in the BG (BG energy factor) and tNAA in the BG (BG neuronal factor).

**Table 5:** Factor loadings assigned according to correlations between total choline (tCho), total N-acetyl-aspartate (tNAA) and total creatine (tCr) within the basal ganglia (BG), midfrontal gray matter (MFGM) and peritrigonal white matter (PWM), when using *varimax* rotation.

	Factor 1	Factor 2	Factor 3	Factor 4	Factor 5
tCho BG	**0.778**	0.146	<0.1	0.476	0.372
tNAA BG	<0.1	0.145	<0.1	0.229	**0.636**
tCr BG	<0.1	0.119	<0.1	**0.954**	0.255
tCho MFGM	**0.680**	<0.1	0.332	<0.1	−0.113
tNAA MFGM	0.181	0.160	**0.707**	−0.139	0.224
tCr MFGM	0.301	0.176	**0.919**	0.153	<0.1
tCho PWM	**0.394**	−0.124	0.170	<0.1	<0.1
tNAA PWM	<0.1	**0.583**	0.124	<0.1	0.367
tCr PWM	<0.1	**0.975**	0.177	0.110	<0.1
	Multi-regional inflammatory factor	PWM axonal factor	MFGM neuronal factor	BG energy factor	BG neuronal factor

The highest loading for each metabolite is shown in bold and factors were named based on metabolites which contributed the greatest loading.

When introducing weighted scores from factor analysis with *varimax* rotation into logistic regression models, the multi-regional inflammatory and PWM axonal factors could distinguish between children with PHIV and children without HIV at the age of 11 years (Table 6). While 4.9 times the odds of HIV infection were associated with a unit increase in the inflammatory factor, there was a 63.0% decrease in the relative odds of HIV infection for a unit increase in the PWM axonal factor (Table 6), when adjusting for other potential confounding variables.

**Table 6:** Logistic regression model investigating the association between HIV status and factor scores for the weighted multi-regional inflammatory factor and peritrigonal white matter (PWM) axonal factor, based on *varimax* rotation, adjusted for confounding variables.

N = 123 (71 PHIV, 52 HIV-)	Odds ratio	95% CI	p-value
Inflammatory factor	**4.877**	**2.296**–**11.732**	**<0.001**
PWM axonal factor	**0.370**	**0.145**–**0.854**	**0.026**
Age at scan	2.213	0.442–12.064	0.341
BG gray matter content	1.033	0.974–1.097	0.277
MFGM gray matter content	0.968	0.867–1.078	0.559
PWM white matter content	0.970	0.916–1.022	0.277
Sex (male)	0.614	0.266–1.397	0.247

PHIV indicates perinatal HIV; HIV- includes children who were HEU and HU; Bold denotes p < 0.05.

**Fig. 4:**
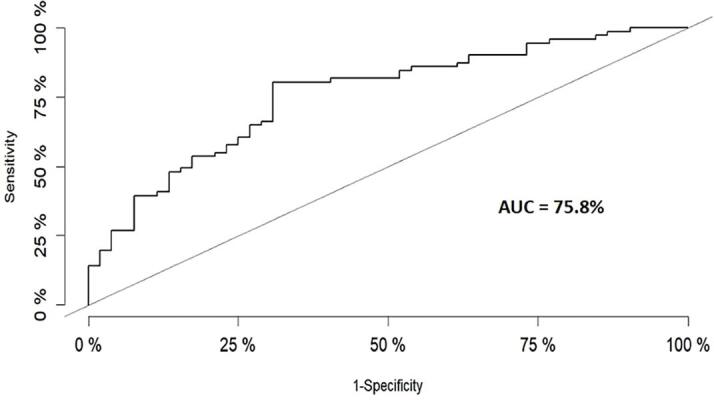
Receiver operating characteristic (ROC) curve for logistic regression model including weighted factor scores based on *varimax* rotation. The area under the curve (AUC) is 75.8%.

A receiver operating characteristic (ROC) curve, defining model sensitivity and specificity, is shown for the logistic regression model assessing the relationship between HIV status and the inflammatory and PWM axonal factor scores (Fig. 4). The capability of the model to predict the outcome (HIV status) was assessed using the area under the curve (AUC) percentage. The final logistic regression model with factor scores based on *varimax* rotation, had an AUC of 75.8%, indicating moderate predictive ability.

## 4 Discussion

In an ongoing neurodevelopmental study, small metabolic changes are observed in specific brain regions of 11-year-old children with HIV after perinatal infection, despite early treatment. The impacts of exposure to HIV and ART are still seen in seronegative children born to WLHIV. Further, we identify patterns of metabolic activity that may differentiate children with PHIV from children without HIV at 11 years.

### 4.1 Restored metabolic activity in the BG of children with PHIV and children who were HEU

Absolute metabolite concentrations, and ratios to creatine, in the BG are similar between children with PHIV and children who were HU, and between children who were HEU and children who were HU. This indicates that harmful effects of perinatal HIV infection and early ART, or exposure, are no longer observed in this region at the age of 11 years, as previously noted at 7 years (Robertson et al., 2018). Early treatment in children with PHIV may prevent long-term damage in this region.

At 9 years lower levels of NAA and Glu were observed for children with PHIV and children who were HEU, while children who were HEU further expressed lower Cr and Cho levels (Robertson et al., 2018). Although we had hypothesised that the levels of BG NAA and Glu would continue to be lower in children with PHIV and children who were HEU, this was not observed at 11 years.

### 4.2 Elevated tCho in the MFGM of children with PHIV

In the MFGM voxel we detect slightly elevated tCho levels in children with PHIV, similar to that observed in the frontal cortex and occipital gray matter of adults with HIV, which is usually associated with an inflammatory response, excessive cell proliferation or an influx of cells (Valcour et al., 2012, Harezlak et al., 2011). Neuroinflammation, during HIV infection, is driven by the release of host cytokines and chemokines by infected macrophages, and in the presence of viral proteins, such as Tat (Valcour et al., 2012, Toborek et al., 2003, Persidsky et al., 2000, McManus et al., 2000). This finding of elevated tCho is supported by elevations in the tCho/tCr ratio in the MFGM.

However, it is believed that elevated Cho alone is insufficient to conclude that inflammation is occurring and an elevation in Ins should also be present (Harezlak et al., 2011). Our finding of elevated absolute Ins in the MFGM that fell just short of conventional levels of significance, provides additional support to our suggestion that there is inflammation in this region.

Elevated tCho/tCr in the frontal gray matter has been observed in previous MRS studies by our group, of a subset of children with PHIV from the CHER trial. At 7 years, children with PHIV had elevated tCho levels in the MFGM, but by the age of 9 years this was no longer seen relative to children who were HU or HEU (unpublished data). As such, we had expected that by 11 years the levels of choline in the MFGM would be unchanged in children with PHIV in comparison to the controls.

The unexpected observation of elevated Cho might indicate that at 11 years children with PHIV return to a similar inflammatory state to that observed at 7 years. However, it should be noted that at 7 years there were 27 controls who were HU in the sample, while at 9 years there were only 18 controls who were HU (unpublished data). Thus, although more children with PHIV contributed data at 9 years, there may have been less statistical power to detect differences in Cho because of the smaller control group. Elevated Cho in the MFGM of children with PHIV may in fact be consistent across the ages at which the children have been scanned, despite the appearance of temporarily restored Cho levels at 9 years.

### 4.3 Neuronal damage in the MFGM of children who were HEU

In this study at 11 years, small reductions in Glu and NAA levels are detected in children who were HEU. As NAA is considered a marker of neuronal integrity, a reduction in gray matter NAA supports the possibility that there may be neuronal damage (Robertson et al., 2018, Harezlak et al., 2011, López-Villegas et al., 1997). Reductions in Glu have previously been suggested to indicate that there may be ART-driven mitochondrial toxicity that impacts the synthesis of Glu in the tricarboxylic acid cycle, or that the uptake of extracellular Glu from the synaptic cleft is not being effectively carried out by the astrocytes (Ernst et al., 2010). It is surprising that we do not see a similar result in children with PHIV, if treatment is in fact having this effect. However, this may be due to greater adherence to ART during pregnancy among the mothers of children who were HEU, and therefore their children being exposed to higher doses of ART in utero.

In an early study of how HIV causes neuronal damage, exposure to the viral envelope protein gp120 was shown to reduce the ability of astrocytes to take up extracellular glutamate (Vesce et al., 1997). In the presence of too much extracellular Glu, excessive neuronal stimulation via N-methyl-D-aspartate receptors can occur, leading to neuronal damage or loss (Ernst et al., 2010, Jiang et al., 2001, Kaul et al., 2001). This may explain how HIV exposure, without infection, could impact Glu uptake in children who were HEU.

A reduction in MFGM Glu has previously been observed at 7 and 9 years in children with PHIV from this cohort, compared to controls who were HU, yet this was not seen for children who were HEU (unpublished data). In the BG region at 9 years, however, lower NAA and Glu concentrations were found for children who were HEU (Robertson et al., 2018).

Thus, at 11 years in children who were HEU, it is possible that glutamate-driven neuronal damage had occurred in the MFGM. It should, however, be noted that the ratios of Glu and NAA to tCr did not differ between children who were HEU and HU, suggesting that the difference between these groups is small and any damage would likely be minor. As the HEU group was smaller than the PHIV group, we had less statistical power to detect differences when comparing them to children who were HU.

### 4.4 Reduction in tNAA and tCr in the PWM

In the peritrigonal white matter, reductions in tNAA and tCr concentrations were detected in children with PHIV. Additionally, lower concentrations of tNAA occurred in children who were HEU in comparison to children who were HU.

In a previous study of children and adolescents with HIV, the ratio of NAA/Cr in parietal white matter was lower in individuals with HIV and encephalopathy than in those without encephalopathy, but not compared to controls (Pavlakis et al., 1995). The authors suggested that reduced WM NAA is an indication of axonal injury (Pavlakis et al., 1995). Although our results do not lead us to the same conclusions as Pavlakis et al. (1995), they suggest that axonal damage in the PWM may also be occurring at 11 years in both children with PHIV and children who were HEU.

White matter damage has previously been reported in this cohort: diffusion tensor imaging studies at the ages of 5 and 7 years found greater radial diffusivity and lower fractional anisotropy (FA) in specific brain regions of children with PHIV, which was proposed to indicate depletion of, or damage to, the myelin sheath (Jankiewicz et al., 2017, Ackermann et al., 2016). At 5 years, the right corticospinal tract contained the areas where this was most noticeable (Ackermann et al., 2016), while at 7 years lower FA was reported in the left inferior fronto-occipital and left inferior longitudinal fasciculi (Jankiewicz et al., 2017). These papers support our observations of anomalies in white matter due to HIV infection.

As Cr provides an internal reference in many MRS studies, reductions in Cr levels are rarely reported. PCr is catabolized to release Cr and phosphate for ATP generation (Gualano et al., 2010), while cyclical activity occurs with the addition of phosphate to Cr in the mitochondria (Steen et al., 2010). Thus, reductions in tCr in the white matter in HIV infection, might indicate a reduction in energy production or energy requirements.

When looking at the impact of treatment interruption in children with PHIV, we found that the reduction in tCr was driven specifically by children for whom treatment had not been interrupted. It is possible that the observation of lower tCr in children with PHIV is related to treatment, rather than HIV infection. ART drugs, particularly nucleoside reverse transcriptase inhibitors, have toxic effects in mitochondria, causing an increased number of mutations in mitochondrial DNA and, therefore impacting respiratory chain activity (Ouyang et al., 2018, Payne et al., 2011, Gingelmaier et al., 2009). This may lead to a lower demand for Cr and PCr in children with PHIV.

Metabolite ratios to tCr in the PWM at 11 years were similar between children with PHIV and children who were HU, and between children who were HEU and HU. In WM brain regions, various studies have previously found elevated Cho/Cr in children with PHIV (Van Dalen et al., 2016, Prado et al., 2011), while a study by Keller et al. (2004) found lower Cho levels in the frontal white matter for children with PHIV. This study, however, found that both tCho/tCr and absolute tCho levels in the PWM were similar between the groups and thus our results neither support the findings of Van Dalen et al., 2016, Prado et al., 2011, nor those of Keller et al. (2004).

The absence of differences in ratios of the various metabolites to tCr in the PWM at 11 years may be because of the differing absolute levels of tCr between children with PHIV and children who were HU - given that the absolute concentration of tNAA differs between these children. Consequently, although Cr is commonly used as a reference metabolite in ^1^H-MRS studies, it may not be a valid reference for studying the effects of HIV within this region of the brain.

The focus of this discussion is on results that have not been adjusted for multiple comparisons. Observed p-values are given and hence readers can see the relative strength of associations. However, if one wishes to use the 5% level of significance as a binary decision maker for significant versus non-significant results, it should be noted that we see more significant findings than could reasonably be considered to occur only by chance, indicating that there are still small metabolic differences at 11 years resulting from perinatal HIV infection or exposure, despite children and mothers receiving ART.

### 4.5 Altered cross-regional metabolic pattern in children with PHIV

The linear regression approach used above, does not consider interregional covariations of a particular metabolite or the intraregional relationships between different metabolites. However, there is reason to expect that response patterns of multiple metabolites would be related, due to complex underlying networks of metabolic pathways (Rae, 2014, Grachev and Apkarian, 2002) - which may not be directly observed.

Factor analysis reduces many correlated variables into a smaller number of latent variables or factors, enabling interesting patterns of metabolic activity to be identified. Adapting the approach of Yiannoutsos et al. (2004) to assess differences in metabolic patterns in children with PHIV vs children without HIV, we identified five factors. Surprisingly, only the multi-regional inflammatory factor grouped a single metabolite, tCho, across all regions. However, NAA and Cr were grouped within the MFGM as well as within the PWM, reflecting an association between these metabolites within both of these regions.

The introduction of weighted factor scores into a logistic regression model showed that the inflammatory factor, driven by tCho in the three regions of interest, and the PWM axonal factor, driven by PWM tCr and tNAA, distinguished between children with and without PHIV at 11 years. We obtained similar results when introducing the weighted scores based on an oblique *promax* rotation (for method see Finch, 2006, Abdi, 2003), and when using a simple scoring approach (as in Yiannoutsos et al., 2004) (results not shown).

Children with PHIV had higher inflammatory factor scores, than children without HIV, because of higher combined concentrations of tCho in the three regions of interest. This result reinforces our findings from linear regression analysis but may also implicate elevated WM Cho in HIV. This tCho elevation may indicate a simultaneous inflammatory response across the brain, in both gray and white matter. A previous study of peripheral cytokine and chemokine responses at 7 years, revealed elevations in proinflammatory cytokines such as IL-1 and IL-6 in a cohort of these children from the CHER trial (Naidoo et al., 2018). Thus, our results at 11 years suggest that, in addition to driving a peripheral inflammatory response, HIV may also drive inflammation in the brain.

Related work in adults, using spectroscopic imaging, similarly found a Cho factor reflecting elevated Cho across deep GM and WM regions that distinguished between patients with HIV and controls (Mohamed et al., 2010). However, this factor did not differentiate between individuals with HIV with and without dementia (Mohamed et al., 2010). Other work by Yiannoutsos et al. (2004) that focused on differences in metabolic patterns between adults with HIV, also found that an inflammatory factor, driven by Cho/Cr and Ins/Cr ratios in centrum semiovale and parietal cortex voxels, did not distinguish between individuals with and without dementia. However, a factor representing elevated NAA/Cr and Cho/Cr in the BG, as well as a factor dependent on NAA/Cr reductions in centrum semiovale and parietal cortex, were strongly associated with dementia in adults with HIV (Yiannoutsos et al., 2004).

At 11 years, lower PWM axonal factor scores, resulting from lower coupled PWM tNAA and tCr concentrations, were found in children with PHIV compared to children without HIV. Again, this reinforces our findings from the linear regression analysis while providing evidence of a possible association between NAA and Cr in WM. Although we only identified one multi-regional factor, Van Dalen et al. (2016) showed strong associations between the levels of several individual metabolites across GM and WM in children with PHIV, as well as relationships between metabolites within GM and WM (Van Dalen et al., 2016).

Since many studies considered ratios to Cr, including the factor analysis study by Yiannoutsos et al. (2004) and the study by Van Dalen et al. (2016), a within-region association of NAA and Cr has not previously been identified in individuals with HIV. In previous spectroscopy studies of healthy adults, several strong intraregional metabolite associations have been found, while few strong interregional correlations have been presented (Waddell et al., 2011, Grachev and Apkarian, 2002). Although these papers suggest that the best means of grouping metabolite activity is by region, we have found some informative patterns by also looking across the regions of interest.

The predictive ability of our logistic regression model, incorporating factor scores, appears to be moderate given an AUC percentage above 75%. Whilst an MRS approach would not necessarily be a good stand-alone diagnostic tool for HIV as metabolic changes can be the outcome of numerous different pathologies, utilising factor analysis and logistic regression could support the identification of biomarkers of HIV infection in the brain. It would, nevertheless, be interesting to investigate the potential in this approach for tracking the prognosis of individuals with HIV, in particular the neurological impacts with increasing disease severity.

### 4.6 Limitations and future work

Of the MRS studies focusing on children with PHIV, the majority are from economically developed countries (Van Dalen et al., 2016, Nagarajan et al., 2012, Gabis et al., 2006, Keller et al., 2004, Pavlakis et al., 1995). Our study, however, provides findings specific to South African children, from a resource-limited setting and with a high burden of HIV.

Although this study provides insight into the metabolic changes occurring in perinatally infected children, a limitation is that we are unable to differentiate between the effects of HIV and ART. Further, we have carried out cross-sectional analysis of the impacts of HIV infection and exposure on metabolic activity in children at 11 years. However, it would be beneficial to carry out longitudinal analysis as this would enable the progression of disease over time, to be studied.

In future we aim to assess the host immune response to HIV in the children from this cohort and how this relates to differences in metabolic activity. We could examine associations with treatment timing and treatment interruption in more depth, and whether inflammation could be the result of viral escape. Additional study could seek to tie in parallel structural and functional studies at 11 years with these spectroscopic findings. This may provide a more holistic picture of the continued effects of perinatal HIV infection and early ART - providing more insight into what these metabolic changes mean in terms of alterations to signalling pathways, structural changes and cognitive impacts.

### 4.7 Conclusion

Based on the findings of this cross-sectional study, the neurometabolic impacts of perinatal HIV infection at 11 years include a slight elevation in tCho in the MFGM region, and a small reduction in tNAA and tCr in the PWM. Differing tCr levels, between children with PHIV and children who were HU, suggest that it may be advisable not to solely rely on metabolite ratios to creatine but to also look at the absolute concentrations of metabolites.

Our results indicate possible inflammation in gray matter and axonal damage in white matter, despite children with PHIV beginning early treatment and most of them (97%) being virally suppressed. An increase in the multi-regional inflammatory factor scores would indicate that inflammation may be a global phenomenon of HIV infection in the brain. However, whether these results can be extrapolated to gray and white matter across the entire brain, or whether these results are specific solely to the regions we assessed, cannot be determined from this study alone. Finally, the implications of these metabolic alterations at a cognitive level, will need to be investigated further.

## Author contributions

Conceptualisation of the study, data collection, methodology and acquisition of funds were carried out by EM, AvdK and BL. AG, FR and FL were involved in processing and analysis of the data. The draft manuscript was written by AG, with review and editing carried out by FR, EM, MH, AvdK, FL, BL, MC and ED.

## Funding

This work was supported by funding from the National Research Foundation of South Africa (Grant Number: 117183, DST-NRF Innovation Masters scholarship); the Masters VC Research Scholarship at the University of Cape Town; UCT VC Interim Funding 2011/2012; the NRF/DST South African Research Chairs Initiative; NRF grant CPRR150723129691; South African Medical Research Council (SAMRC); US National Institute of Allergy and Infectious Diseases (NIAID) (CIPRA network, Grant U19 AI53217); and NIH grants (R01 HD099846, R01 DC015984, R01HD071664, R21MH096559 and R21MH108346).

## Declaration of Competing Interest

The authors declare that they have no known competing financial interests or personal relationships that could have appeared to influence the work reported in this paper.

## References

[b0005] Abdi, H., 2003. Factor rotations in factor analyses. In: Encyclopedia for Research Methods for the Social Sciences. Sage, Thousand Oaks, CA, pp. 792–795.

[b0010] Ackermann C., Andronikou S., Laughton B., Kidd M., Dobbels E., Innes S., van Toorn R., Cotton M. (2014). White matter signal abnormalities in children with suspected HIV-related neurologic disease on early combination antiretroviral therapy. Pediatr. Infect. Dis. J..

[b0015] Ackermann C., Andronikou S., Saleh M.G., Laughton B., Alhamud A.A., van der Kouwe A., Kidd M., Cotton M.F. (2016). Early antiretroviral therapy in HIV-infected children is associated with diffuse white matter structural abnormality and corpus callosum sparing. Am. J. Neuroradiol..

[b0020] Alexander G.E., DeLong M.R., Strick P.L. (1986). Parallel organization of functionally segregated circuits linking basal ganglia and cortex. Annu. Rev. Neurosci..

[b0025] Bonelli R.M., Cummings J.L. (2007). Frontal-subcortical circuitry and behavior. Dialogues Clin. Neurosci..

[b0030] Booth J.R., Wood L., Lu D., Houk J.C., Bitan T. (2007). The role of the basal ganglia and cerebellum in language processing. Brain Res..

[b0035] Brass M., Von Cramon D.Y. (2002). The role of the frontal cortex in task preparation. Cereb. Cortex.

[b0040] Casey B.J., Jones R.M., Hare T.A. (2008). The adolescent brain. Ann. N. Y. Acad. Sci..

[b0045] Cotton M.F., Violari A., Otwombe K., Panchia R., Dobbels E., Rabie H., Josipovic D., Liberty A. (2013). Early time-limited antiretroviral therapy versus deferred therapy in South African infants infected with HIV: results from the children with HIV early antiretroviral (CHER) randomised trial. Lancet.

[b0050] De Cock K.M., Fowler M.G., Mercier E., De Vincenzi I., Saba J., Hoff E., Alnwick D.J., Rogers M. (2000). Prevention of mother-to-child HIV transmission in resource-poor countries: translating research into policy and practice. JAMA.

[b0055] Diamond A. (2002). Normal development of prefrontal cortex from birth to young adulthood: cognitive functions, anatomy, and biochemistry. Principles Frontal Lobe Func..

[b0060] Ernst T., Kreis R., Ross B.D. (1993). Absolute quantitation of water and metabolites in the human brain. I. Compartments and water. J. Magn. Reson., Ser B.

[b0065] Ernst T., Jiang C.S., Nakama H., Buchthal S., Chang L. (2010). Lower brain glutamate is associated with cognitive deficits in HIV patients: a new mechanism for HIV-associated neurocognitive disorder. J. Magn. Reson. Imaging.

[b0070] Finch H. (2006). Comparison of the performance of varimax and promax rotations: factor structure recovery for dichotomous items. J. Educ. Meas..

[b0075] Gabis L., Belman A., Huang W., Milazzo M., Nachman S. (2006). Clinical and imaging study of human immunodeficiency Virus-1—infected youth receiving highly active antiretroviral therapy: pilot study using magnetic resonance spectroscopy. J. Child Neurol..

[b0080] Gingelmaier A., Grubert T.A., Kost B.P., Setzer B., Lebrecht D., Mylonas I., Mueller-Hoecker J., Jeschke U. (2009). Mitochondrial toxicity in HIV type-1-exposed pregnancies in the era of highly active antiretroviral therapy. Antivir Ther..

[b0085] Grachev I.D., Apkarian A.V. (2002). Multi-chemical networking profile of the living human brain: potential relevance to molecular studies of cognition and behavior in normal and diseased brain. J. Neural Transm..

[b0090] Graybiel A.M. (1995). Building action repertoires: memory and learning functions of the basal ganglia. Curr. Opin. Neurobiol..

[b0095] Gualano B., Artioli G.G., Poortmans J.R., Junior A.H.L. (2010). Exploring the therapeutic role of creatine supplementation. Amino Acids.

[b0100] Harezlak J., Buchthal S., Taylor M., Schifitto G., Zhong J., Daar E.S., Alger J., Singer E. (2011). Persistence of hiv− associated cognitive impairment, inflammation and neuronal injury in era of highly active antiretroviral treatment. AIDS (London, England)..

[b0105] Helms G. (2008). The principles of quantification applied to in vivo proton MR spectroscopy. Eur. J. Radiol..

[b0110] Holmes M.J., Robertson F.C., Little F., Randall S.R., Cotton M.F., van der Kouwe A.JW., Laughton B., Meintjes E.M. (2017). Longitudinal increases of brain metabolite levels in 5–10 year old children. PLoS ONE.

[b0115] Horn P.S., Feng L., Li Y., Pesce A.J. (2001). Effect of outliers and nonhealthy individuals on reference interval estimation. Clin. Chem..

[b0120] Jankiewicz M., Holmes M.J., Taylor P.A., Cotton M.F., Laughton B., Der Kouwe V., Andre J.W., Meintjes E.M. (2017). White matter abnormalities in children with HIV infection and exposure. Front. Neuroanat..

[b0125] Jiang Z., Piggee C., Heyes M.P., Murphy C., Quearry B., Bauer M., Zheng J., Gendelman H.E. (2001). Glutamate is a mediator of neurotoxicity in secretions of activated HIV-1-infected macrophages. J. Neuroimmunol..

[b0130] Joos B., Fischer M., Kuster H., Pillai S.K., Wong J.K., Böni J., Hirschel B., Weber R. (2008). HIV rebounds from latently infected cells, rather than from continuing low-level replication. Proc. Natl. Acad. Sci..

[b0135] Kaiser H.F. (1958). The varimax criterion for analytic rotation in factor analysis. Psychometrika.

[b0140] Kaul M., Garden G.A., Lipton S.A. (2001). Pathways to neuronal injury and apoptosis in HIV-associated dementia. Nature.

[b0145] Keller M.A., Venkatraman T.N., Thomas A., Deveikis A., LoPresti C., Hayes J., Berman N., Walot I. (2004). Altered neurometabolite development in HIV-infected children Correlation with neuropsychological tests. Neurology.

[b0150] Kerr S.J., Puthanakit T., Vibol U., Aurpibul L., Vonthanak S., Kosalaraksa P., Kanjanavanit S., Hansudewechakul R. (2014). Neurodevelopmental outcomes in HIV-exposed-uninfected children versus those not exposed to HIV. AIDS Care.

[b0155] Laughton B., Cornell M., Grove D., Kidd M., Springer P.E., Dobbels E., van Rensburg A.J., Violari A. (2012). Early antiretroviral therapy improves neurodevelopmental outcomes in infants. AIDS (London, England).

[b0160] Laughton B., Cornell M., Kidd M., Springer P.E., Dobbels E.F.M., Rensburg A.J.V., Otwombe K., Babiker A. (2018). Five year neurodevelopment outcomes of perinatally HIV-infected children on early limited or deferred continuous antiretroviral therapy. J. Int. AIDS Soc..

[b0165] López-Villegas D., Lenkinski R.E., Frank I. (1997). Biochemical changes in the frontal lobe of HIV-infected individuals detected by magnetic resonance spectroscopy. Proc. Natl. Acad. Sci..

[b0170] Madhi S.A., Adrian P., Cotton M.F., McIntyre J.A., Jean-Philippe P., Meadows S., Nachman S., Käyhty H. (2010). Effect of HIV infection status and anti-retroviral treatment on quantitative and qualitative antibody responses to pneumococcal conjugate vaccine in infants. J. Infect. Dis..

[b0175] Malee K.M., Tassiopoulos K., Huo Y., Siberry G., Williams P.L., Hazra R., Smith R.A., Allison S.M. (2011). Mental health functioning among children and adolescents with perinatal HIV infection and perinatal HIV exposure. AIDS Care.

[b0180] Mbugua K., Holmes M., Cotton M., Ratai E., Little F., Hess A., Dobbels E., Van der Kouwe A. (2016). HIV-associated CD4+/CD8+ depletion in infancy is associated with neurometabolic reductions in the basal ganglia at age 5 years despite early antiretroviral therapy. Aids.

[b0185] McManus C.M., Weidenheim K., Woodman S.E., Nunez J., Hesselgesser J., Nath A., Berman J.W. (2000). Chemokine and chemokine-receptor expression in human glial elements: induction by the HIV protein, Tat, and chemokine autoregulation. Am. J. Pathol..

[b0190] Mellins C.A., Elkington K.S., Leu C., Santamaria E.K., Dolezal C., Wiznia A., Bamji M., Mckay M.M. (2012). Prevalence and change in psychiatric disorders among perinatally HIV-infected and HIV-exposed youth. AIDS Care.

[b0195] Mohamed M.A., Lentz M.R., Lee V., Halpern E.F., Sacktor N., Selnes O., Barker P.B., Pomper M.G. (2010). Factor analysis of proton MR spectroscopic imaging data in HIV infection: metabolite-derived factors help identify infection and dementia. Radiology.

[b0200] Naidoo, S., Veldsman, K., Cotton, M.F., Glashoff, R.H., July 2018. Persistence of myeloid cell-associated inflammation in HIV-infected children after 8 years on early initiated therapy - the key role players in HIV persistence? International AIDS Society (IAS). Amsterdam, Netherlands (Poster and Oral).

[b0205] Nagarajan R., Sarma M.K., Thomas M.A., Chang L., Natha U., Wright M., Hayes J., Nielsen-Saines K. (2012). Neuropsychological function and cerebral metabolites in HIV-infected youth. J. Neuroimmune Pharmacol..

[b0210] Ouyang Y., Wei F., Qiao L., Liu K., Dong Y., Guo X., Wang S., Pang L. (2018). Mitochondrial DNA mutations accumulated in HIV-1-infected children who have an excellent virological response when exposed to long-term antiretroviral therapy. J. Antimicrob. Chemother..

[b0215] Parazzini C., Baldoli C., Scotti G., Triulzi F. (2002). Terminal zones of myelination: MR evaluation of children aged 20–40 months. Am. J. Neuroradiol..

[b0220] Paul R.H., Ernst T., Brickman A.M., Yiannoutsos C.T., Tate D.F., Cohen R.A., Navia B.A. (2008). Relative sensitivity of magnetic resonance spectroscopy and quantitative magnetic resonance imaging to cognitive function among nondemented individuals infected with HIV. J. Int. Neuropsychol. Soc..

[b0225] Pavlakis, S.G., Lu, D., Frank, Y., Bakshi, S., Pahwa, S., Barnett, T.A., Porricolo, M.E., Gould, R.J. et al., 1995. Magnetic resonance spectroscopy in childhood AIDS encephalopathy. Pediatr. Neurol. 12(4): 277–282. DOI:088789949500048K [pii]. 10.1016/0887-8994(95)00048-k 7546001

[b0230] Payne B.A., Wilson I.J., Hateley C.A., Horvath R., Santibanez-Koref M., Samuels D.C., Price D.A., Chinnery P.F. (2011). Mitochondrial aging is accelerated by anti-retroviral therapy through the clonal expansion of mtDNA mutations. Nat. Genet..

[b0235] Persidsky Y., Zheng J., Miller D., Gendelman H.E. (2000). Mononuclear phagocytes mediate blood-brain barrier compromise and neuronal injury during HIV-1-associated dementia. J. Leukoc. Biol..

[b0240] Prado P.T., Escorsi-Rosset S., Cervi M.C., Santos A.C. (2011). Image evaluation of HIV encephalopathy: a multimodal approach using quantitative MR techniques. Neuroradiology.

[b0245] Provencher S.W. (2001). Automatic quantitation of localized in vivo1H spectra with LCModel. NMR Biomed..

[b0250] R Core Team, 2018. R: A Language and Environment for Statistical Computing. Available: https://www.R-project.org.

[b0255] Rae C.D. (2014). A guide to the metabolic pathways and function of metabolites observed in human brain 1 H magnetic resonance spectra. Neurochem. Res..

[b0260] Randall S.R., Warton C.M., Holmes M.J., Cotton M.F., Laughton B., van der Kouwe A.JW., Meintjes E.M. (2017). Larger subcortical gray matter structures and smaller corpora callosa at age 5 years in HIV infected children on early ART. Front. Neuroanat..

[b0265] Robertson F.C., Holmes M.J., Cotton M.F., Dobbels E., Little F., Laughton B., Der Kouwe V., André J.W., Meintjes E.M. (2018). Perinatal HIV infection or exposure is associated with low N-acetylaspartate and Glutamate in Basal Ganglia at age 9 but not 7 years. Front. Hum. Neurosci..

[b0270] Robertson K., Liner J., Meeker R.B. (2012). Antiretroviral neurotoxicity. J. Neurovirol..

[b0275] Soares D.P., Law M. (2009). Magnetic resonance spectroscopy of the brain: review of metabolites and clinical applications. Clin. Radiol..

[b0280] Steen C., Wilczak N., Hoogduin J.M., Koch M., De Keyser J. (2010). Reduced creatine kinase B activity in multiple sclerosis normal appearing white matter. PLoS ONE.

[b0285] Stringer E.M., Ekouevi D.K., Coetzee D., Tih P.M., Creek T.L., Stinson K., Giganti M.J., Welty T.K. (2010). Coverage of nevirapine-based services to prevent mother-to-child HIV transmission in 4 African countries. J. Am. Med. Assoc..

[b0290] Toborek M., Lee Y.W., Pu H., Malecki A., Flora G., Garrido R., Hennig B., Bauer H. (2003). HIV-Tat protein induces oxidative and inflammatory pathways in brain endothelium. J. Neurochem..

[b0295] Toich J.T., Taylor P.A., Holmes M.J., Gohel S., Cotton M.F., Dobbels E., Laughton B., Little F. (2018). Functional connectivity alterations between networks and associations with infant immune health within networks in HIV infected children on early treatment: a study at 7 years. Front. Hum. Neurosci..

[b0300] UNAIDS, 2019. UNAIDS data 2019. Available: https://www.unaids.org/en/resources/documents/2019/2019-UNAIDS-data [Aug 9, 2019].

[b0305] Valcour V., Chalermchai T., Sailasuta N., Marovich M., Lerdlum S., Suttichom D., Suwanwela N.C., Jagodzinski L. (2012). Central nervous system viral invasion and inflammation during acute HIV infection. J. Infect. Dis..

[b0310] Van Dalen Y.W., Blokhuis C., Cohen S., Ter Stege J.A., Teunissen C.E., Kuhle J., Kootstra N.A., Scherpbier H.J. (2016). Neurometabolite alterations associated with cognitive performance in perinatally HIV-infected children. Medicine.

[b0315] van der Kouwe A.JW., Benner T., Salat D.H., Fischl B. (2008). Brain morphometry with multiecho MPRAGE. NeuroImage.

[b0320] Van Rie A., Mupuala A., Dow A. (2008). Impact of the HIV/AIDS epidemic on the neurodevelopment of preschool-aged children in Kinshasa, Democratic Republic of the Congo. Pediatrics.

[b0325] Vesce S., Bezzi P., Rossi D., Meldolesi J., Volterra A. (1997). HIV-1 gp120 glycoprotein affects the astrocyte control of extracellular glutamate by both inhibiting the uptake and stimulating the release of the amino acid. FEBS Lett..

[b0330] Violari A., Cotton M.F., Gibb D.M., Babiker A.G., Steyn J., Madhi S.A., Jean-Philippe P., McIntyre J.A. (2008). Early antiretroviral therapy and mortality among HIV-infected infants. N. Engl. J. Med..

[b0335] Waddell K.W., Zanjanipour P., Pradhan S., Xu L., Welch E.B., Joers J.M., Martin P.R., Avison M.J. (2011). Anterior cingulate and cerebellar GABA and Glu correlations measured by 1H J-difference spectroscopy. Magn. Reson. Imaging.

[b0340] Warszawski J., Tubiana R., Le Chenadec J., Blanche S., Teglas J., Dollfus C., Faye A., Burgard M. (2008). Mother-to-child HIV transmission despite antiretroviral therapy in the ANRS French Perinatal Cohort. Aids.

[b0345] WHO, 2013. WHO | 7.1.4 When to start ART in children. Available: http://www.who.int/hiv/pub/guidelines/arv2013/art/statartchildren/en/ [Jan 31, 2018].

[b0350] WHO (2017). WHO | Guidelines for managing advanced HIV disease and rapid initiation of antiretroviral therapy. Available: http://www.who.int/hiv/pub/guidelines/advanced-HIV-disease/en/ [Feb.

[b0355] Wilkinson I.D., Lunn S., Miszkiel K.A., Miller R.F., Paley M.N., Williams I., Chinn R.J., Hall-Craggs M.A. (1997). Proton MRS and quantitative MRI assessment of the short term neurological response to antiretroviral therapy in AIDS. J. Neurol. Neurosurg. Psychiatry.

[b0360] Yiannoutsos C.T., Ernst T., Chang L., Lee P.L., Richards T., Marra C.M., Meyerhoff D.J., Jarvik J.G. (2004). Regional patterns of brain metabolites in AIDS dementia complex. NeuroImage.

