# Supplementary material for: MRS suggests multi-regional inflammation and white matter axonal damage at 11 years following perinatal HIV infection
Source: Neuroimage Clin. 2020 Nov 19;28:102505. doi: 10.1016/j.nicl.2020.102505 (PMC7721646; doi:10.1016/j.nicl.2020.102505)
Supplement: Supplementary data 1 [file mmc1.docx]

**MRS suggests multi-regional inflammation and white matter axonal damage at 11 years following perinatal HIV infection.**

**Supplementary results**

S.1 Voxel composition

**Supplementary table 1:** The mean gray matter (GM), white matter (WM) and cerebral spinal fluid content (± standard deviation) in the basal ganglia (BG), midfrontal gray matter (MFGM) and peritrigonal white matter (PWM), according to HIV status groups.

|  |  | PHIV | HEU | HU | F (p) |
| --- | --- | --- | --- | --- | --- |
| GM (%) | BG | 59.76±8.41 | 57.66±7.63 | 56.03±7.57 | 2.50 (0.09) |
|  | MFGM | 86.03±4.21 | 85.10±7.00 | 86.45±4.01 | 0.62 (0.54) |
|  | PWM | 20.21±13.77 | 20.63±11.57 | 20.85±9.35 | 0.03 (0.97) |
| WM (%) | BG | 40.05±8.50 | 42.30±7.63 | 43.86±7.44 | 2.62 (0.08) |
|  | MFGM | 2.19±1.25 | 2.97±1.96 | 2.60±1.31 | **3.26 (0.04)** |
|  | PWM | 79.48±13.88 | 78.75±11.45 | 78.89±9.41 | 0.05 (0.95) |
| CSF (%) | BG | 0.19±1.12 | 0.04±0.10 | 0.11±0.41 | 0.34 (0.71) |
|  | MFGM | 11.76±4.45 | 11.93±7.67 | 10.87±4.05 | 0.38 (0.68) |
|  | PWM | 0.31±0.51 | 0.62±1.11 | 0.26±0.45 | 2.72 (0.07) |

S.2 Regression analysis models

**S.2.1 Linear regression analysis models:**

Y = β_0_ + β_1_X_1_ + β_2_X_2_ + β_3_X_3_ + β_4_X_4_, weight=(1/Z)

where Y=metabolite concentration, X1=HIV status (PHIV or HEU vs HU), X2=Age at scan, X3=Sex, X4=gray/white matter content, Z= standard deviation for metabolite concentration.

**S.2.2 Logistic regression analysis models:**

logit(P) = β_0_ + β_1_X_1_ + β_2_X_2_ + β_3_X_3_ + β_4_X_4_ + β_5_X_5_ + β_6_X_6_ + β_7_X_7_

where P=Probability of HIV infection, X1=Inflammatory factor, X2=PWM axonal factor, X3=Age at scan, X4=Sex, X5=BG gray matter content, X6=MFGM gray matter content and X7= PWM white matter content.
